# Parvovirus B19 infection preceding the diagnosis of childhood myelodysplastic syndrome with low blasts: a case report

**DOI:** 10.3389/fonc.2026.1818574

**Published:** 2026-06-05

**Authors:** Leah Klingel, Stefanie Huber, Irith Baumann, Stephan Schwarz-Furlan, Almut Meyer-Bahlburg, Holger Lode

**Affiliations:** 1 Department of Pediatric Hematology and Oncology, University Medical Center Greifswald, Greifswald, Germany; 2 Institute of Pathology, Kaufbeuren-Ravensburg, Germany; 3 Department of Pediatrics, Rheumatology and Immunology, University Medical Center Rostock, Rostock, Germany; 4 Department of Pediatrics, Rheumatology and Immunology, University Medical Center Greifswald, Greifswald, Germany

**Keywords:** cMDS-LB, HSCT = hematopoietic stem cell transplant, myelodsyplastic syndromes, parvovirus B 19, refractory cytopenia of childhood

## Abstract

Parvovirus B19 (PVB19) infection is a recognized cause of transient bone marrow suppression and pure red cell aplasia; however, pancytopenia with hypocellular marrow and multilineage dysplasia is uncommon in children and poses a diagnostic challenge. We report the case of a previously healthy 7-year-old girl who presented with pancytopenia during acute PVB19 infection, confirmed by high viral loads in peripheral blood, cerebrospinal fluid, and bone marrow. Initial bone marrow examination demonstrated hypocellularity with multilineage dysplasia but did not meet criteria for myelodysplastic syndrome. Comprehensive evaluation excluded autoimmune disease, primary immunodeficiency, inherited bone marrow failure syndromes, and known pediatric MDS predisposition genes. Serial follow-up revealed persistent hypocellularity and evolving dysplasia, ultimately establishing the diagnosis of childhood MDS with low blasts (cMDS-LB). The patient underwent successful allogeneic hematopoietic stem cell transplantation with stable full donor chimerism and hematologic recovery. Post-transplant, she experienced episodes of febrile pneumonitis accompanied by transient increases in PVB19 DNA levels, followed by a gradual decline with persistent low-level PVB19 DNAemia at last follow-up (day +618). This case highlights that PVB19 infection may temporally coincide with, reveal, or contribute to an evolving pediatric myelodysplastic process rather than directly causing it.

## Introduction

Parvovirus B19 (PVB19) infection is a well-established cause of transient bone marrow suppression and pure red cell aplasia, particularly in patients with underlying hematologic disorders or immunodeficiency (1–3). PVB19 is the only erythroparvovirus known to be pathogenic to humans and the etiologic agent of erythema infectiosum. Owing to the high affinity of its capsid proteins for the P antigen expressed on erythroid progenitor cells, PVB19 selectively targets erythroid progenitor cells, leading to suppression of erythropoiesis and, in some cases, broader bone marrow dysfunction (4).

From a clinical perspective, PVB19 infection primarily affects patients with hemolytic anemia and hematological malignancies, typically manifesting as pure red cell aplasia (2). Severe bone marrow failure has also been described in immunodeficient states, including leukemia and HIV infection (2, 3, 5). Episodes of severe but transient cytopenia following PVB19 infection have additionally been reported in otherwise healthy older children, underscoring the variable hematologic impact of this virus (6).

In immunocompetent individuals, acute PVB19 infection follows a characteristic and usually self-limiting course. High-level viremia typically persists for less than one week and declines with the development of virus-specific immunoglobulin M (IgM), which remains detectable for approximately 8–10 weeks, followed by lifelong immunoglobulin G (IgG)–mediated immunity (3, 4). By adulthood, serological evidence of prior PVB19 infection is present in up to 80% of the population (7). In addition to humoral immunity, virus-specific cellular immune responses play an important role in viral clearance (8).

In immunocompromised patients, impaired viral clearance can lead to persistent PVB19 infection and chronic viral carriage, often presenting with unexplained or severe anemia and marked reticulocytopenia (9, 10). However, PVB19 DNA may also persist in bone marrow of immunocompetent individuals—with or without symptoms—highlighting that PCR positivity alone does not necessarily indicate active disease (11).

Findings in the peripheral blood in PVB19 infection typically demonstrate predominant erythroid suppression, although transient leukopenia and thrombocytopenia may occur, reflecting intact myeloid and megakaryocytic lineages, alongside variable red blood cell abnormalities such as anisocytosis, poikilocytosis, and occasional nucleated erythrocytes. Bone marrow examination characteristically reveals erythroid maturation arrest with accumulation of early precursors, including giant pronormoblasts (“lantern cells”) with vacuolated cytoplasm, cytoplasmic projections, and eosinophilic nuclear inclusions (3, 7).

While erythroid hypoplasia is a common feature, hemophagocytosis and more extensive bone marrow involvement have been reported in selected cases (12, 13). In contrast, pancytopenia accompanied by hypocellular bone marrow and multilineage dysplasia is distinctly uncommon in PVB19 infection and raises concern for alternative or concomitant bone marrow failure syndromes, including myelodysplastic syndrome (14, 15). Distinguishing transient virus-associated bone marrow suppression from evolving clonal hematopoietic disease is particularly challenging in pediatric patients and often requires careful longitudinal assessment.

In this context, we report the case of a previously healthy 7-year-old girl with age-appropriate development who presented with pancytopenia during acute PVB19 infection and subsequently evolved to childhood MDS with low blasts (cMDS-LB).

## Case

The patient was referred to our clinic after incidental detection of pancytopenia during evaluation of gastrointestinal symptoms. One week before presentation, she had a single febrile episode (maximum 38.5 °C) with headache and rhinitis. She had no relevant past medical history and was described as previously healthy. Initial laboratory testing confirmed pancytopenia with anemia, thrombocytopenia, leukopenia, and neutropenia.

**Figure 1:**
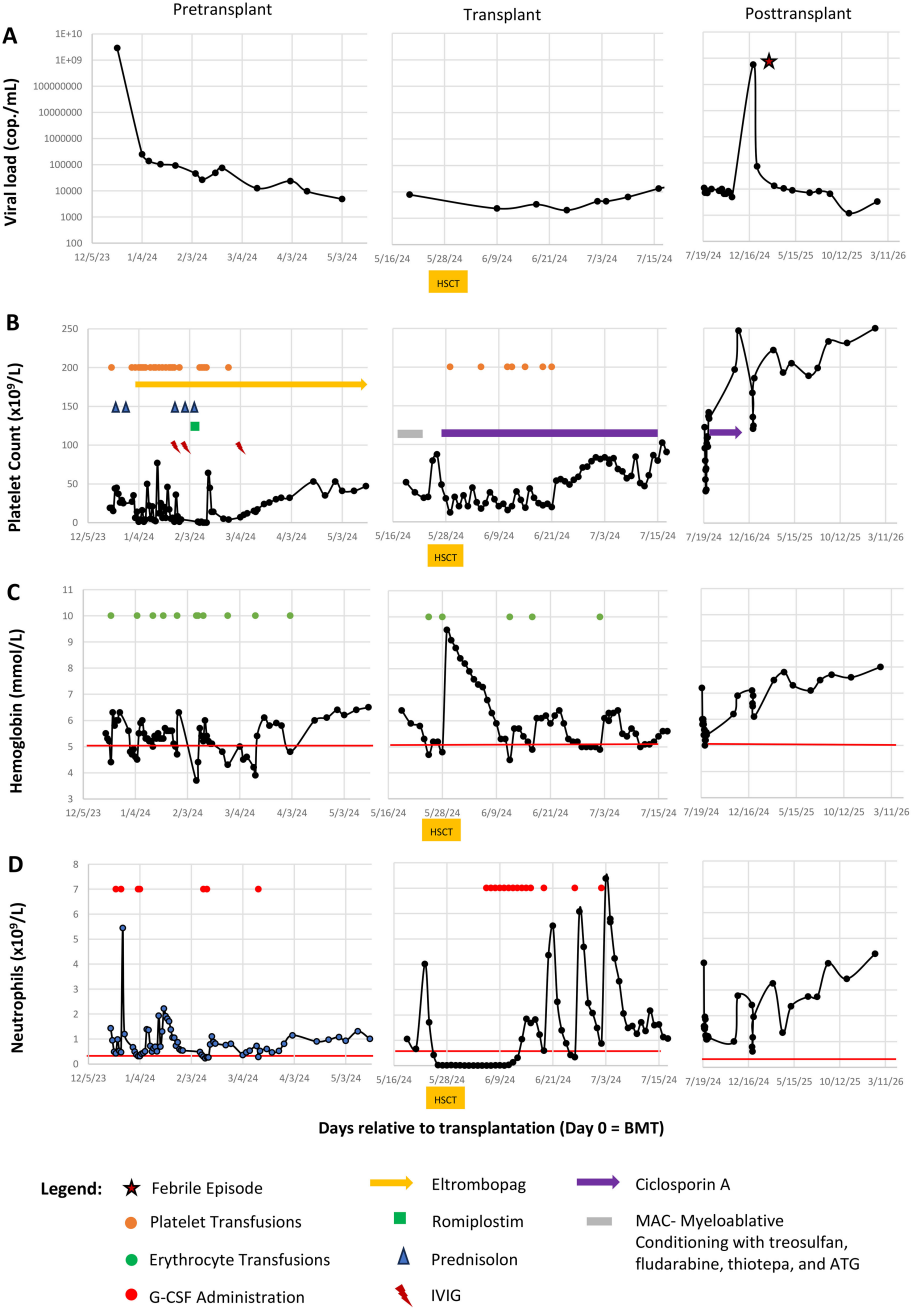
Longitudinal virologic and hematologic course before and after allogeneic hematopoietic stem cell transplantation (HSCT). **(A)** Parvovirus B19 DNA levels demonstrate an initially high viral load with gradual decline, a transient increase during a febrile episode in December 2024, and sustained suppression after HSCT, with low-level persistence until the last follow-up. Hematologic parameters are shown relative to the transplant date (day 0 = 28 May 2024). The myeloablative conditioning (MAC) regimen consisted of treosulfan, fludarabine, thiotepa, and ATG; ciclosporin was administered from day -1 to +100. **(B)** Platelet counts (×10^9^/L) show severe therapy-refractory thrombocytopenia prior to transplantation and durable engraftment thereafter. Platelet transfusion thresholds (red horizontal line) were <20 ×10^9^/L pre-transplant and <30 ×10^9^/L during transplantation. **(C)** Hemoglobin levels demonstrate persistent pre-transplant anemia followed by gradual recovery after HSCT without further red blood cell transfusions (transfusion threshold: hemoglobin <5 mmol/L; red line). Green circles indicate erythrocyte transfusions. **(D)** Absolute neutrophil counts (×10^9^/L) reveal recurrent pre-transplant neutropenia with stabilization post-transplant. Before HSCT, repeated therapies-including red blood cell and platelet transfusions, intravenous immunoglobulin (IVIG), corticosteroids, thrombopoietin receptor agonists, and G-CSF-produced only transient responses, consistent with an underlying bone marrow failure syndrome. See legend.

Serologic testing demonstrated acute PVB19 infection, with positive IgM, negative IgG, and a high plasma viral load by quantitative PCR (2,929,600,000 copies/mL) (**Figure 1A**). Quantitative PCR of cerebrospinal fluid also showed a high viral load (3,027,520 copies/mL). Testing for other viral pathogens, including HSV, VZV, CMV, EBV, enterovirus, and adenovirus, was negative, and no bacterial or fungal pathogens were identified.

**Figure 2:**
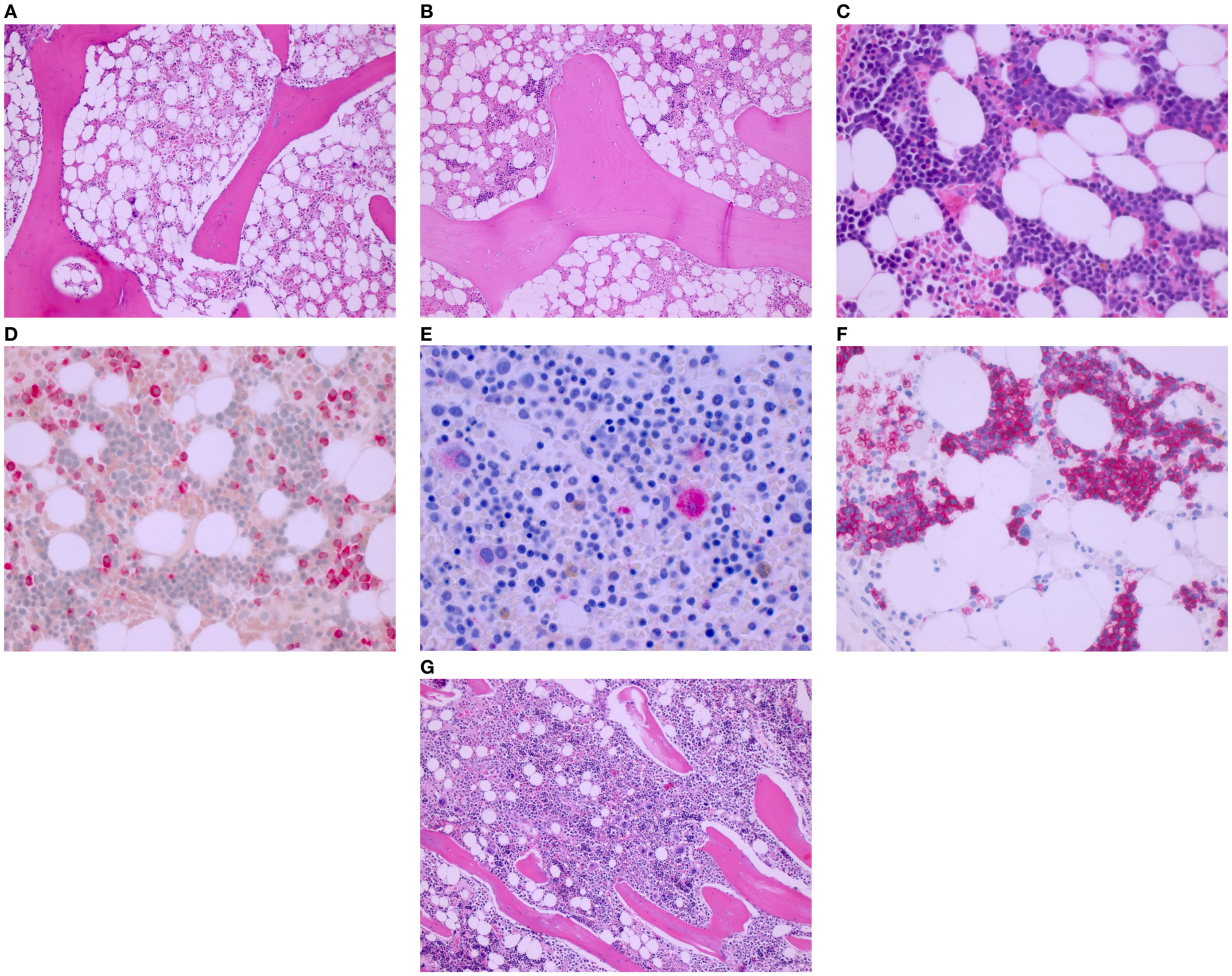
Serial bone marrow histopathology demonstrating the evolution from acute PVB19-associated marrow suppression to childhood MDS with low blasts and subsequent post-transplant marrow recovery. **(A)** Initial bone marrow biopsy demonstrating marked hypocellularity and patchy residual hematopoiesis H&E staining of the initial bone marrow biopsy (×100) demonstrating marked hypocellularity with increased adipose tissue and patchy residual hematopoiesis, including focal erythropoiesis and granulopoiesis, without excess blasts. **(B)** Follow-up bone marrow biopsy demonstrating persistent marked hypocellularity and ongoing marrow failure. Second bone marrow biopsy obtained during longitudinal follow-up demonstrating persistent marked hypocellularity with increased adipose tissue and patchy residual hematopoiesis, consistent with ongoing marrow failure (H&E, ×100). **(C)** Third bone marrow biopsy demonstrating persistent hypocellularity and evolving multilineage dysplasia Third longitudinal bone marrow biopsy demonstrating persistent hypocellularity with increased adipose tissue, dysplastic multilineage hematopoiesis, reduced granulopoiesis, and atypical megakaryocytic forms, consistent with evolving childhood MDS with low blasts (H&E, ×400). **(D)** CAE staining of the third bone marrow biopsy demonstrating severe granulopoietic hypoplasia CAE staining of the third bone marrow biopsy demonstrating markedly reduced granulopoiesis with sparse distribution of granulocytic precursor cells, consistent with severe granulopoietic hypoplasia (×400). **(E)** CD61 immunostaining demonstrating dysplastic megakaryocytic maturation in the third bone marrow biopsy CD61 immunostaining of the third bone marrow biopsy demonstrating dysplastic megakaryocytes, including atypical mononuclear forms, consistent with abnormal megakaryocytic maturation (×400). **(F)** CD71 immunostaining demonstrating expanded erythropoiesis in the third bone marrow biopsy CD71 immunostaining of the third bone marrow biopsy demonstrating marked expansion of erythropoiesis with identifiable mitotic figures, indicating increased erythroid proliferative activity (×400). **(G)** Post-transplant bone marrow biopsy demonstrating restoration of marrow cellularity and multilineage hematopoiesis Bone marrow biopsy obtained on day +100 after allogeneic HSCT demonstrating restoration of marrow cellularity with reconstituted multilineage hematopoiesis and no evidence of excess blasts (H&E, ×100).

Initial bone marrow biopsy demonstrated marked hypocellularity with patchy residual erythropoiesis and granulopoiesis, without evidence of excess blasts (**Figure 2A**). Smears demonstrated erythroid hyperplasia with dysplastic features, hypoplastic granulopoiesis with dysplasia and left shift, and absent megakaryopoiesis. Bone marrow quantitative PCR showed a markedly elevated Parvovirus B19 viral load (2,872,960,000 copies/mL). At this stage, diagnostic criteria for childhood MDS with low blasts (cMDS-LB) were not fulfilled, and there was no evidence of severe aplastic anemia. Targeted next-generation sequencing for pediatric MDS predisposition genes (GATA2, RUNX1, SAMD9, and SAMD9L) revealed no pathogenic variants.

An extensive diagnostic workup excluded alternative causes of pancytopenia. Autoimmune disorders were ruled out. Immunological evaluation demonstrated normal immunoglobulin levels and preserved vaccine responses, without evidence of primary immunodeficiency. Investigations for inherited bone marrow failure syndromes, including Fanconi anemia and telomere biology disorders, were unrevealing. Conventional cytogenetic analysis showed a normal karyotype. Screening for paroxysmal nocturnal hemoglobinuria identified only a very small clone, considered clinically insignificant. Extended targeted genetic testing for genes associated with immune dysregulation and inherited bone marrow failure syndromes, including NBN, TCN2, SLC46A1, ERCC6L2, SRP72, ATR, WRAP53, TERT, FAS, FASLG, CASP10, CASP8, PRKCD, IL2RA, CTLA4, KRAS, FADD, STAT3, STAT1, CARD11, DEF6, RELA, NFKB1, and LRBA, did not identify any clinically relevant pathogenic or likely pathogenic variants.

Although the initial presentation was compatible with PVB19–associated marrow suppression, the subsequent course raised concern for an underlying marrow-intrinsic process. Cytopenias persisted and progressed despite supportive and immunomodulatory treatment. Despite multiple interventions, thrombocytopenia and anemia progressively worsened, with platelet counts ultimately declining to zero while the patient remained clinically stable (**Figures 1B, C**). This profound lack of hematologic recovery was paralleled by progressive neutropenia that also remained refractory to therapy, arguing against isolated peripheral immune destruction (**Figure 1D**).

Repeat bone marrow examinations at eight and twelve weeks demonstrated persistent and evolving multilineage abnormalities (**Figures 2B–F**). The first follow-up marrow remained markedly hypocellular, with pronounced granulocytic hypoplasia, dysplastic maturation, and absent megakaryopoiesis. The second follow-up showed increased overall cellularity, but persistent dysplasia across multiple lineages, including abnormal erythroid maturation, ongoing granulopoietic hypoplasia, and reduced, morphologically abnormal megakaryocytes. Blast counts remained low throughout.

Based on persistent cytopenias, serial marrow findings with evolving multilineage dysplasia, and exclusion of alternative inherited and acquired causes, the diagnosis of childhood MDS with low blasts was established in consultation with the national reference center. A suitable unrelated donor was identified, and the patient underwent allogeneic hematopoietic stem cell transplantation following myeloablative conditioning. Graft-versus-host disease prophylaxis consisted of ciclosporin A, initiated on day −1 and continued through day +100. Hematopoietic engraftment was successfully achieved, and serial chimerism analyses demonstrated stable full donor chimerism. Following HSCT, PVB19 DNA levels showed an overall decline but remained detectable by quantitative PCR. Bone marrow cellularity subsequently normalized (**Figure 2G**). Intercurrent infectious episodes were accompanied by transient increases in viral load; however, these were not associated with sustained graft dysfunction or persistent cytopenias. At last follow-up, the patient remained clinically stable with durable hematologic recovery despite persistent low-level detectable PVB19 DNAemia.

## Discussion

Parvovirus B19 is a well-recognized cause of transient bone marrow suppression and pure red cell aplasia, particularly in immunocompromised hosts (16). In immunocompetent children, infection is typically self-limiting; however, persistent infection may occur in the setting of impaired humoral immunity or broader immunocompromise, leading to chronic anemia and reticulocytopenia (17). In contrast, pancytopenia with hypocellular bone marrow and dysplastic features is distinctly uncommon in PVB19 infection. It can closely mimic clonal bone marrow disorders, which makes distinguishing between severe virus-associated marrow suppression and myelodysplastic syndromes particularly challenging in pediatric patients (3, 15).

This case illustrates the diagnostic complexity of such presentation. At initial evaluation, the patient exhibited profound cytopenias in conjunction with extremely high PVB19 DNA levels in peripheral blood, cerebrospinal fluid, and bone marrow. The first bone marrow examination revealed hypocellularity with erythroid aplasia, dysplastic granulopoiesis and megakaryopoiesis, and mild hemophagocytosis—findings that, in the context of overwhelming viremia, were initially interpreted as consistent with Parvovirus B19–associated marrow failure (**Figure 2A**).

However, the subsequent clinical course proved decisive. Despite supportive and immunomodulatory therapy, the patient developed progressive, treatment-refractory pancytopenia, including G-CSF–refractory neutropenia and therapy-resistant thrombocytopenia. Serial bone marrow examinations at eight and twelve weeks demonstrated worsening hypocellularity, absent or markedly reduced megakaryopoiesis, and evolving multilineage dysplasia—features no longer compatible with isolated viral marrow suppression. This further supports that persistent detection of PVB19 alone cannot account for the observed progressive marrow failure.

Because dysplastic changes may occur as reactive and potentially reversible phenomena during viral infections, including PVB19 (15, 18), a diagnosis of cMDS-LB at initial presentation would have been premature, particularly in the absence of excess blasts or defining cytogenetic or molecular abnormalities. Instead, longitudinal clinical and morphological assessment was pursued. The persistence and progression of marrow failure, persistent low blast counts together with increasingly overt multilineage dysplasia, ultimately fulfilled diagnostic criteria for cMDS-LB. Within the current WHO 2022 framework, such cases are classified as cMDS-LB (formerly RCC), a category in which diagnosis relies heavily on serial marrow evaluation and the exclusion of reactive mimics. Contemporary pediatric MDS risk stratification further integrates marrow cellularity, cytogenetic abnormalities (notably high-risk lesions such as monosomy 7), and germline predisposition; in our patient, marked hypocellularity was present, while cytogenetic analysis was normal and no germline predisposition was identified (19). Recent work has highlighted that pediatric MDS is biologically distinct from adult disease, characterized by a higher prevalence of hypocellular marrow and a genetic landscape enriched for RAS-pathway alterations and chromosome 7 abnormalities, rather than the epigenetic mutations commonly seen in adults (20). This evolving understanding of pediatric MDS biology further supports the need for longitudinal evaluation in diagnostically ambiguous hypocellular pediatric marrow disorders. These features, together with a suspected immune-mediated component and relatively fewer cytogenetic abnormalities, complicate early diagnosis and require careful distinction from aplastic anemia and transient post-infectious marrow suppression (21, 22). Accordingly, repeated bone marrow evaluations were essential in this patient to distinguish potentially reversible reactive marrow changes from evolving clonal hematopoietic disease.

A comprehensive evaluation for inherited bone marrow failure syndromes was also essential, as disorders such as Fanconi anemia and telomere biology disorders may present without classical phenotypic features and can manifest in late childhood with progressive pancytopenia and hypocellular marrow (23, 24). Identification of these entities has major therapeutic implications, particularly with respect to tolerance of conditioning regimens prior to HSCT. In this patient, the absence of syndromic features, a normal karyotype, normal telomere length, and the lack of pathogenic variants in known pediatric MDS predisposition genes (including GATA2, RUNX1, SAMD9, and SAMD9L) made an inherited predisposition unlikely and supported standard-risk transplant planning.

In parallel, viral kinetics provided important contextual information: following the initial peak, PVB19 DNA levels declined but remained detectable after transplantation, with transient increases during febrile episodes and intercurrent infections—a pattern described in immunocompromised patients that is more consistent with impaired viral clearance or reactivation than with reinfection (9, 25–27). Notably, these fluctuations were not associated with sustained cytopenias or impaired graft function, and complete donor chimerism was maintained. Together, these findings suggest that persistent detection of PVB19 DNA after HSCT should be interpreted cautiously, as PCR positivity may reflect delayed clearance of residual viral genomes rather than clinically relevant active infection.

These findings raise the question of whether PVB19 represents a coincidental finding, a disease modifier, or a trigger for clonal evolution. PVB19 exhibits marked tropism for erythroid progenitor cells via the P antigen and can induce apoptosis, cell-cycle arrest, and inflammatory cytokine responses, thereby imposing marrow stress and immune dysregulation (4, 28, 29). Although no direct causal relationship between PVB19 infection and myelodysplastic syndromes has been established, available evidence supports an associative rather than causal role, with viral infection potentially revealing, modifying, or temporally coinciding with an evolving underlying marrow disorder (15, 18, 29). Once the diagnosis of cMDS-LB was established, timely allogeneic hematopoietic stem cell transplantation—the only established curative option—was essential, and the favorable engraftment kinetics and manageable infectious complications in our patient support early transplantation after diagnostic clarification (30).

Reports describing the coexistence of PVB19 infection and pediatric MDS are scarce. Small case series have documented patients with persistent PVB19 infection and MDS who failed prolonged IVIG therapy and ultimately required HSCT, with variable but generally favorable outcomes (31). Similar to these reports, our patient showed persistent and therapy-refractory cytopenias requiring definitive treatment by allogeneic HSCT. However, unlike cases in which the distinction between infection-related marrow suppression and clonal disease remained uncertain, serial marrow examinations in our patient demonstrated progressive multilineage dysplasia and persistent hypocellularity, ultimately supporting classification within the pediatric low-blast MDS spectrum. Conversely, isolated reports describe transient myelodysplastic or JMML-like features during acute PVB19 infection that resolved spontaneously, highlighting the virus’s capacity to mimic clonal myeloid disease (32).

In addition, our case demonstrated prolonged persistence of PVB19 DNA after HSCT despite full donor chimerism and sustained hematologic recovery. Although viral persistence or reactivation early after transplantation has been reported in immunocompromised patients (10, 26, 27), persistence beyond 365 days after HSCT appears to be uncommon. Importantly, the prolonged DNAemia in our patient was not associated with impaired hematopoiesis or graft dysfunction, supporting the interpretation that persistent PCR positivity may reflect delayed clearance of residual viral genomes rather than clinically relevant active infection.

## Conclusion

This case highlights the diagnostic complexity of severe cytopenias occurring during acute PVB19 infection in children and underscores the importance of longitudinal clinical and morphological assessment. While PVB19 can cause profound but transient bone marrow suppression, persistent and progressive cytopenias with evolving multilineage dysplasia should prompt repeated bone marrow evaluation to exclude an underlying clonal disorder. Our findings support the concept that PVB19 may function as a disease modifier or contributing factor rather than a direct cause of pediatric myelodysplastic disease. Importantly, persistent PVB19 DNAemia after successful allogeneic hematopoietic stem cell transplantation does not necessarily indicate active infection or impaired graft function.

## Data Availability

The original contributions presented in the study are included in the article/supplementary material. Further inquiries can be directed to the corresponding author.
